# Molecular profiling of biliary tract cancers reveals distinct genomic landscapes between circulating and tissue tumor DNA

**DOI:** 10.1186/s40164-023-00470-7

**Published:** 2024-01-08

**Authors:** Clémence Astier, Carine Ngo, Léo Colmet-Daage, Virginie Marty, Olivia Bawa, Claudio Nicotra, Maud Ngo-Camus, Antoine Italiano, Christophe Massard, Jean-Yves Scoazec, Cristina Smolenschi, Michel Ducreux, Antoine Hollebecque, Sophie Postel-Vinay

**Affiliations:** 1 ERC Chromatin Remodeling, DNA Repair and Epigenetics Laboratory, INSERM U981, Gustave Roussy, Villejuif, France; 2 Université Paris-Saclay, Université Paris-Sud XI, Faculté de Médicine, Le Kremlin Bicêtre, France; 3 Department of Pathology, Gustave Roussy, Villejuif, France; 4 INSERM US23, CNRS UAR 3655, AMMICa, Experimental and Translational Pathology Platform, Gustave Roussy, Villejuif, France; 5 Drug Development Department (DITEP), Gustave Roussy – Cancer Campus, Villejuif, France; 6 Department of Early Phase Trial Unit, Institut Bergonié Comprehensive Cancer Centre, Bordeaux, France; 7 Faculty of Medicine, University of Bordeaux, Bordeaux, France; 8 INSERM U1030, Molecular Radiotherapy, Gustave Roussy, Université Paris-Saclay, Paris, France; 9 Department of Pathology and Laboratory Medicine, Translational Research Laboratory and Biobank, AMMICA, INSERM US23/CNRS UMS3655, Gustave Roussy, Villejuif, France; 10 Université Paris-Saclay, Gustave Roussy, INSERM, Dynamique des Cellules Tumorales (U-1279), Villejuif, France; 11 University College of London Cancer Institute, London, UK; 12 Institut Gustave Roussy, 114 rue Edouard Vaillant, 94805 Villejuif, France

**Keywords:** Biliary tract cancer, Cholangiocarcinoma, Molecular landscape, Liquid biopsy, Chromatin remodeling

## Abstract

Biliary tract cancers (BTCs) are heterogeneous malignancies with dismal prognosis due to tumor aggressiveness and poor response to limited current therapeutic options. Tumor exome profiling has allowed to successfully establish targeted therapeutic strategies in the clinical management of cholangiocarcinoma (CCA). Still, whether liquid biopsy profiling could inform on BTC biology and patient management is unknown. In order to test this and generate novel insight into BTC biology, we analyzed the molecular landscape of 128 CCA patients, using a 394-gene NGS panel (Foundation Medicine). Among them, 32 patients had matched circulating tumor (ct) DNA and tumor DNA samples, where both samples were profiled. In both tumor and liquid biopsies, we identified an increased frequency of alterations in genes involved in genome integrity or chromatin remodeling, including *ARID1A (15%)*, *PBRM1 (9%)*, and *BAP1 (14%),* which were validated using an in-house-developed immunohistochemistry panel. ctDNA and tumor DNA showed variable concordance, with a significant correlation in the total number of detected variants, but some heterogeneity in the detection of actionable mutations. *FGFR2* mutations were more frequently identified in liquid biopsies, whereas *KRAS* alterations were mostly found in tumors. All *IDH1* mutations detected in tumor DNA were also identified in liquid biopsies. These findings provide novel insights in the concordance between the tumor and liquid biopsies genomic landscape in a large cohort of patients with BTC and highlight the complementarity of both analyses when guiding therapeutic prescription.

**Supplementary Information:**

The online version contains supplementary material available at 10.1186/s40164-023-00470-7.

**To the editor,**

Incidence and mortality rates of biliary tract cancers (BTC) are rising [1] and most patients present with advanced disease, where standard therapies bring limited benefit [2]. Genomic profiling has allowed the identification of recurrent molecular alterations, leading to successful precision medicine-based therapies (e.g., FGFR or IDH1 inhibitors). Yet, most patients still do not benefit from targeted therapy, due to the absence of actionable alteration or lack of available molecular profile. Lesion accessibility, limited material and tumor heterogeneity represent hurdles to tumor molecular profiling. By contrast, circulating tumor DNA (ctDNA) analysis is minimally invasive, feasible in all patients and possibly recapitulates the molecular landscape of multiple lesions. However, ensuring that alterations identified in liquid biopsies are representative of the ones present in tumors is necessary.

To explore the concordance between molecular alterations in tumor and ctDNA, we characterized the genomic landscape of 128 BTC and 32 matched ctDNA samples using a targeted 394-gene panel (Fig. 1A, B).

**Fig. 1:**
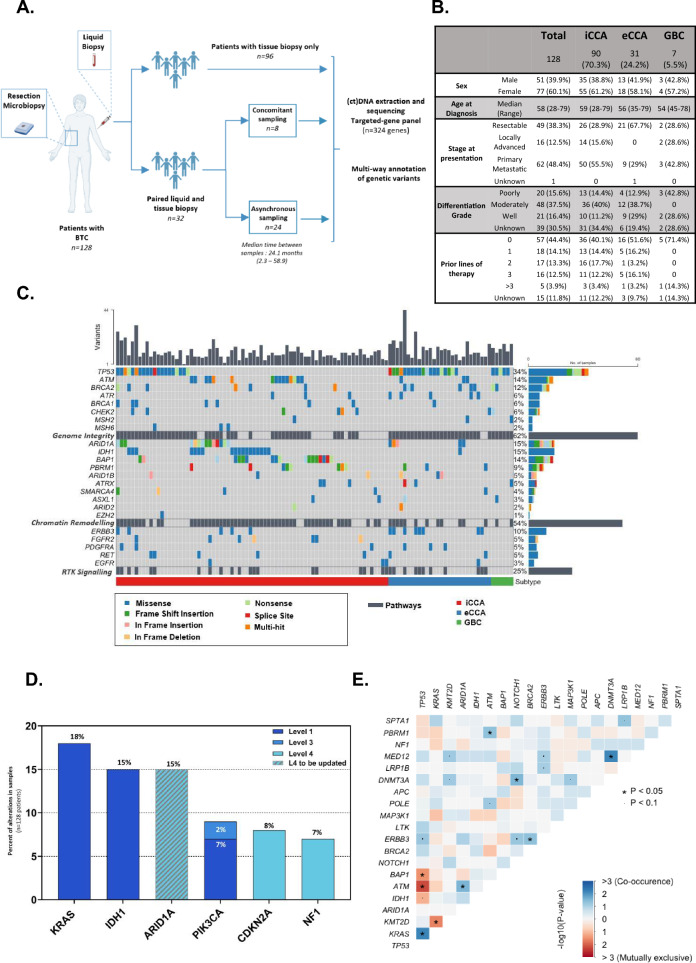
Cholangiocarcinoma harbor frequent DNA repair and chromatin remodeling alterations. **A** Flowchart of patients and samples characterization. **B** Clinico-pathological features of patients with CCA. Prior lines of therapy describe the number of treatment lines received prior to sample collection for molecular characterization; *iCCA* intrahepatic CCA. *eCCA* extrahepatic CCA. **C** Oncoplot of mutations detected in tumor biopsies in 128 patients. Significantly mutated genes are listed vertically in decreasing order and hierarchized by functional category. Colored boxes indicate alteration categories observed in each gene and tumor. **D** Frequency of targetable mutations for OncoKB level 1–4 alterations with a > 5% prevalence. Level 1 = FDA-approved drugs, Level 3 = Clinical Evidence, Level 4 = Biological Evidence. *ARID1A* status should be updated to level 1–2 according to latest FDA approval of Tulmimetostat. **E** Somatic interactions plot for the 20 most frequently altered genes. Colored boxes show mutual exclusivity and co-occurring alterations between two genes (*p < 0.05)

## Molecular features

Overall, 1357 genomic alterations were detected in 333 genes of the 128 tumor samples. Most frequently altered genes were *TP53*, *KRAS*, *KMT2*D, *ARID1A*, *IDH1*, *ATM* and *BAP1.* Among 71 tumors for which microsatellite stability (MS) status was assessed, 69 were MS-Stable and two were MS-Instable (Additional file 1: Fig. S1A). Most frequently altered genes were involved in “Genome integrity” (62%) and “Chromatin remodeling” (54%), notably subunits of the SWI/SNF chromatin remodeling complex (35% of cases, including *ARID1A, PBRM1*, *ARID1B*, *SMARCA4* and *ARID2)*. Alterations in these pathways were significantly associated with a higher number of variants (Additional file 1: Fig. S1B). Interestingly, we found that, as previously described for *FGFR* and *IDH1* mutations [3], *BAP1* alterations were restricted to iCCA (Fig. 1C).

When focusing on clinically actionable alterations, *KRAS* (G12/V/C, OncoKB level 1), *IDH1* (level 1) and *ARID1A* (level 1–2) [4, 5] were most frequently altered (18%, 15% and 15% of patients, respectively; Fig. 1D). *KRAS* and *TP53* alterations tended to co-occur (p < 0.05), like *DNMT3A* and *MED12*. By contrast, *TP53* mutations were mutually exclusive with *ATM* and *BAP1* alterations, as *KMT2D* and *KRAS* (Fig. 1E).

## Genomic landscape of tissue versus liquid biopsies

**Fig. 2:**
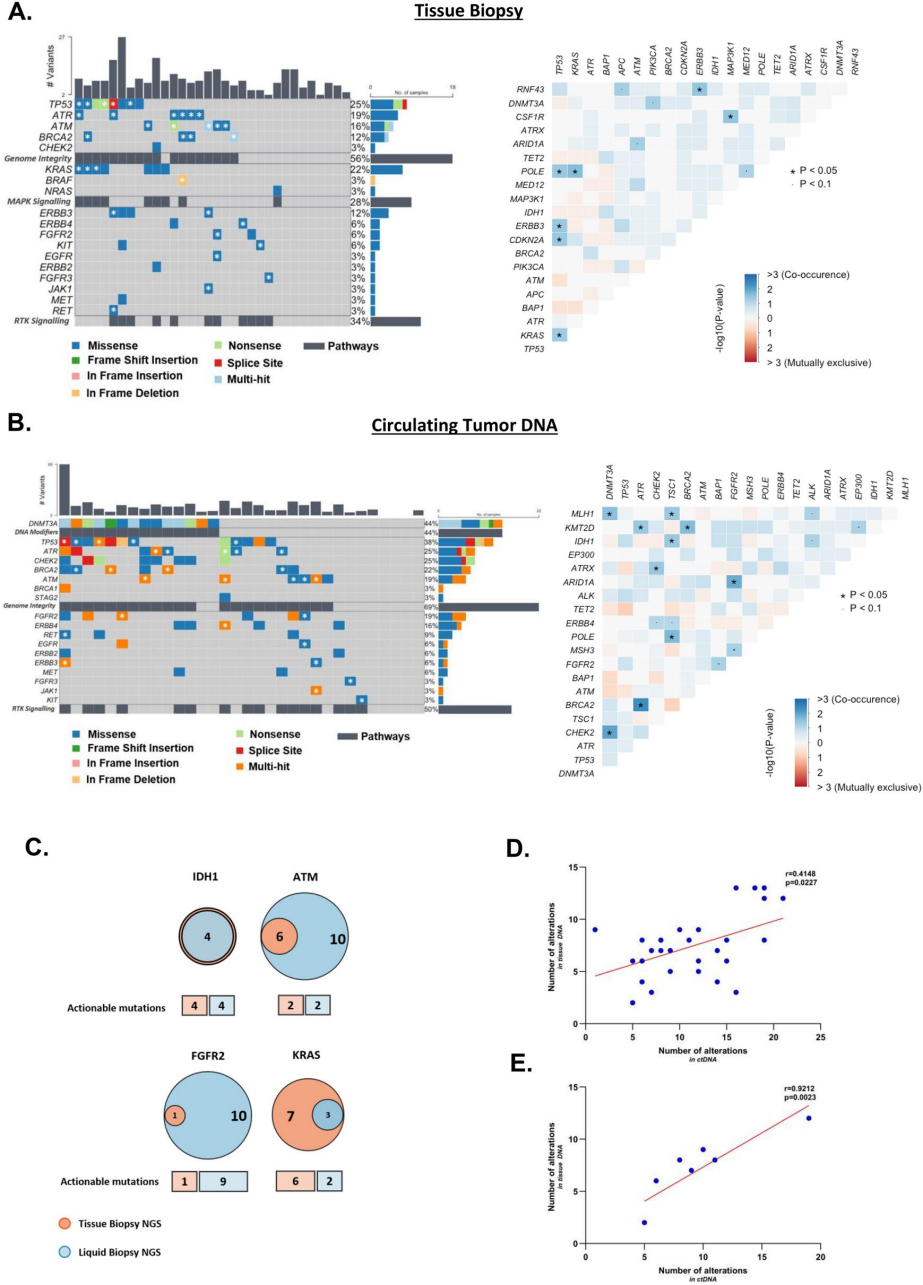
ctDNA and tumor DNA show variable concordance in the detection of molecular alterations. **A**, **B** Oncoplot of mutations detected in tumor (**A**) or liquid (**B**) biopsies in 32 patients with paired samples available. Left panel: white stars represent mutations that were found both in the tumor and in ctDNA. Right panels show somatic interactions of the top 20 altered genes in each sample type. **C** Venn Diagram showing the conservation of *IDH1*, *ATM*, *FGFR2* and *KRAS* alterations between ctDNA and tissue biopsies; boxes below the Venn diagram depict the proportion of actionable mutations. **D** Scatter plot showing the correlation between the number of alterations in ctDNA and tissue DNA in all tumor samples (n = 30, after exclusion of the two outlier patients with MSI-H/MMRd tumors; Spearman correlation score r = 0.4148, p-val = 0.0227, two-tailed t-test). **E** Scatter plot showing the correlation between the number of alterations in ctDNA and tumor samples in patients with concomitant samplings only (n = 8; Spearman correlation score r = 0.9212, p-val = 0.0023, two-tailed t-test)

We next compared molecular alterations in ctDNA versus tumor tissue in a subset of 32 patients with paired samples, including eight with concomitant and 24 with sequential sampling (Additional file 1: Fig. S2A). We identified multiple alterations that were more frequent in liquid biopsies: *DNMT3A* (44% vs 6%), *TP53* (38% vs 25%), *ATR* (25% vs 19%) and *CHEK2* (25% vs 3%). Such discrepancies could result from clonal hematopoiesis of indeterminate potential (CHIP), intra- or inter-tumor heterogeneity, temporal heterogeneity or, low tumor content of the biopsy and should therefore be interpreted with caution. Conversely, *KRAS* mutations were found in 22% of tumor samples, but only in 9% of liquid biopsies. The somatic interactome also showed different patterns: based on tissue analysis, *TP53* alterations co-occurred with *POLE*, *ERBB3*, *CDKN2A* and *KRAS* mutations; this was not observed in plasma samples, where *CHEK2* alterations co-occurred with *DNMT3A* and *ATRX* mutations, potentially linked to CHIP (Fig. 2A, B).

When focusing on clinically actionable alterations, all *IDH1* and *ATM* mutations were conserved in tumor and liquid biopsies. Interestingly, 8/9 *FGFR2* actionable alterations were only detected in liquid biopsies. This may result from the polyclonal secondary mutations following FGFR2-inhibitors therapy [6], since four out of the five patients with actionable alterations received pemigatinib or futibatinib, and progressed at the biopsy timepoint (Additional file 1: Fig. S2B). By contrast, four out of six actionable *KRAS* mutations were only identified in tissue samples (Fig. 2C).

A higher number of ctDNA alterations was found in heavily pre-treated patients, potentially reflecting increased tumor genetic complexity over time and exposure to treatments (Additional file 1: Fig. S3G). No correlation was identified with any other clinical parameter (histotype, metastatic burden, sex, age, differentiation, grade, stage at diagnosis; Additional file 1: Fig. S3 A–H). Noteworthy, the number of alterations found in ctDNA and tumor DNA was significantly correlated, especially in patients with concomitant tumor and ctDNA sampling (Spearman correlation r = 0.9212, p-val = 0.0023 two-tailed t-test; Fig. 2D, E, Additional file 1: Fig. S4).

## Epigenomic alterations

Since deleterious mutations in SWI/SNF or *BAP1* detected in > 30% of this 128-patient cohort are potentially actionable [5, 7, 8], it is crucial to reliably identify them. Still, the pathogenicity of SWI/SNF genes alterations remains vastly unknown. We therefore optimized an immunohistochemistry panel to measure ARID1A, PBRM1, SMARCA4 and SMARCB1 protein expression. ARID1A expression was lost in all *ARID1A*-mutant cases as well as in one *ARID1A*-wild-type *PBRM1*-mutant case. PBRM1 expression was decreased in 5/6 *PBRM1*-mutant samples, and was completely lost in two *BAP1*-mutant cases. Importantly, alteration in SWI/SNF subunit was not anti-correlated with H3K27me3 or EZH2 expression, highlighting the challenge to detect the SWI/SNF-PCR2 epigenetic antagonism in tumors [9] (Additional file 1: Fig. S5).

## Conclusion

In conclusion, molecular profiling of 128 BTC showed frequent alterations in DNA repair and chromatin remodeling factors, notably in SWI/SNF subunits, which increases the proportion of patients with actionable mutations beyond *FGFR2* and *IDH1*. This dataset represents the second largest series of molecular landscape comparison between ctDNA and tumor tissue biopsies in BTC [10, 11]. Limitations of our study include its retrospective and multi-centric nature, the limited sample size, and the heterogeneous patients’ characteristics. Still, we found a significantly positive correlation between the number of alterations detected in matched tumor-liquid biopsy samples, and alterations detected in liquid biopsies were overall concordant with the ones found in tumor tissue. Importantly, discrepancies were also observed notably in actionable alterations, potentially due to spatial or temporal heterogeneity in tumor sampling, or to CHIP-associated false positive [12]. This overall highlights the complementarity of both analyses when guiding therapeutic prescription.

### Supplementary Information

**Additional file 1: Figure S1.** Molecular landscape of cholangiocarcinoma. **A**: Unsupervised oncoplot of alterations landscape in tumor biopsies in 128 patients, where significantly altered genes are listed vertically in decreasing order of prevalence. Colored boxes indicate alteration categories observed in each gene and tumor. MSS status is specified in the lower bar (Red: Not Tested; Blue: MSS, Green: MSI). **B**: Number of variants per sample according to the presence of an alteration in genome integrity- or chromatin-remodeling- related genes; All patients were assessed (n = 128); left panel: alterations in genes involved in genome integrity (p-val < 0.0001, two-tailed Mann–Whitney test); right panel: alterations in genes involved in chromatin remodeling (n = 128; p-val = 0.0322, two-tailed Mann–Whitney test). **Figure S2.** Intermediate treatments between tissue and liquid biopsies in asynchronously sampled patients. **A**: Swimmers’ plot representing the number of lines and type of therapy received by the patients between two asynchronous tissue and liquid biopsies. Colors indicate the type of therapy (chemotherapy, targeted therapy and immunotherapy) and each bar represents a distinct patient. **B**: Initial pre-screening was performed in tissue biopsy, where FGFR2-fusions or –single mutations were detected in these patients (n = 5). Following treatment with either Futibatinib or Pemigatinib (FGFR2 inhibitors), additional liquid biopsy realized at disease progression revealed multi-hit alterations in four of these patients, potentially related to selection pressure on the main driver. **Figure S3.** ctDNA alterations burden according to clinical features. **A**, **B**: Bar plots showing the absence of association between ctDNA alteration burden and BTC histotype (**A**) and metastatic burden (**B**). A: Left panel: all patients (n = 32), with the eCCA outlier corresponding to the patient with an MSI-H tumor (p-val = 0.8649, two-tailed Mann–Whitney test); right panel: patients without the one with MSI-H tumor (p-val = 0.8402, two-tailed Mann–Whitney test). (B). Left panel includes the outlier patient with MSI-H tumor (n = 32; p-val = 0.6469, Kruskal–Wallis test); right panel excluded the outlier patient with MSI-H tumor (n = 31; p-val = 0.7276, Kruskal–Wallis test). **C**–**F:** Other plots show the absence of significant association between ctDNA alterations and sex (C), age at diagnosis (D), differentiation state (E) and stage at diagnosis (F). **G:** Association between the number of prior treatment lines and ctDNA alterations (p-val = 0.0023, Kruskal–Wallis test). **H:** Number of tissue DNA alterations according to the sample type (biopsy versus surgical specimen); without the outlier patient with MSI-H tumor (n = 31; p-val = 0.7276, Kruskal–Wallis test). Abbreviations: BTC: Biliary Tract Cancer; eCCA: extrahepatic CCA, iCCA: intrahepatic CCA; GBC: Gallbladder Cancer; R: Resectable; LA: Locally Advanced; PM: Primary Metastatic. **Figure S4.** Correlations between number of alterations in ctDNA and tumor samples. **A:** All tumor samples are shown (n = 32; Spearman correlation score r = 0.3387, p-val = 0.0579; two-tailed t-test). **B**: Correlation between number of alterations in ctDNA and tumor samples in patients with asynchronous samplings only. Outlier patients with MSI-H/MMRd tumors were excluded (n = 22; Spearman correlation score r = 0.3005, p-val = 0. 1743, two-tailed t-test). **Figure S5.** Data reveal positive correlation between ARID1A/PBRM1 and BAP1/PBRM1 expression. **A:** Table showing *ARID1A*, *PBRM1* and *BAP1 pathogenic* alterations found in 14 patients. Variants were annotated using ANNOVAR. Protein expression for each sample was evaluated by immunohistochemistry. The heatmap summarizes the expression level for each protein according to the presence or absence of mutations. Percentages of positive cells for each marker were evaluated by a senior pathologist. Abbreviations: LOE: Loss of expression; NA: Not available; WT: Wild Type; **B:** Data extracted from Cancer Cell Line Encyclopedia (CCLE)—and available on DepMap Portal—show positive correlations between ARID1A/PBRM1 and BAP1/PBRM1 expression. Among 1451 cell lines evaluated, ARID1A and PBRM1 (left panel) showed a strong positive association, with Pearson’s correlation scores of 0.673 and 0.601 across all cancer lineages and in BTCs lineages respectively (p-val < 0.001). Likewise, PBRM1 and BAP1 (right panel) showed a positive correlation in pan-cancer analyses (Pearson = 0.612, p-val < 0.001) and in BTCs (Pearson = 0.642, p-val < 0.001). **C:** The heatmap summarizes expression level for histone mark H3K27me3 and EZH2 in WT, *PBRM1*, *ARID1A* and *BAP1*-altered CCA cases. No obvious correlation between EZH2 and H3K27me3 levels could be detected; similarly, no anti-correlation was evidenced between SW/SNF subunit loss and H3K27me3 or EZH2 expression in our cohort.

## Data Availability

Data set and sequencing data used and/or analyzed during the current study are available from the corresponding author on reasonable request. Data presented in Additional file 1: Fig. S1C are available on DepMap (https://depmap.org/portal/interactive/).
