# Supplementary material for: Molecular profiling of biliary tract cancers reveals distinct genomic landscapes between circulating and tissue tumor DNA
Source: Exp Hematol Oncol. 2024 Jan 8;13:2. doi: 10.1186/s40164-023-00470-7 (PMC10775454; doi:10.1186/s40164-023-00470-7)
Supplement: Supplementary file 1 — Additional file 1: Figure S1. Molecular landscape of cholangiocarcinoma. A: Unsupervised oncoplot of alterations landscape in tumor biopsies in 128 patients, where significantly altered genes are listed vertically in decreasing order of prevalence. Colored boxes indicate alteration categories observed in each gene and tumor. MSS status is specified in the lower bar (Red: Not Tested; Blue: MSS, Green: MSI). B: Number of variants per sample according to the presence of an alteration in genome integrity- or chromatin-remodeling- related genes; All patients were assessed (n = 128); left panel: alterations in genes involved in genome integrity (p-val < 0.0001, two-tailed Mann–Whitney test); right panel: alterations in genes involved in chromatin remodeling (n = 128; p-val = 0.0322, two-tailed Mann–Whitney test). Figure S2. Intermediate treatments between tissue and liquid biopsies in asynchronously sampled patients. A: Swimmers’ plot representing the number of lines and type of therapy received by the patients between two asynchronous tissue and liquid biopsies. Colors indicate the type of therapy (chemotherapy, targeted therapy and immunotherapy) and each bar represents a distinct patient. B: Initial pre-screening was performed in tissue biopsy, where FGFR2-fusions or –single mutations were detected in these patients (n = 5). Following treatment with either Futibatinib or Pemigatinib (FGFR2 inhibitors), additional liquid biopsy realized at disease progression revealed multi-hit alterations in four of these patients, potentially related to selection pressure on the main driver. Figure S3. ctDNA alterations burden according to clinical features. A, B: Bar plots showing the absence of association between ctDNA alteration burden and BTC histotype (A) and metastatic burden (B). A: Left panel: all patients (n = 32), with the eCCA outlier corresponding to the patient with an MSI-H tumor (p-val = 0.8649, two-tailed Mann–Whitney test); right panel: patients without the one with [file 40164_2023_470_MOESM1_ESM.pptx]

## Slide 1
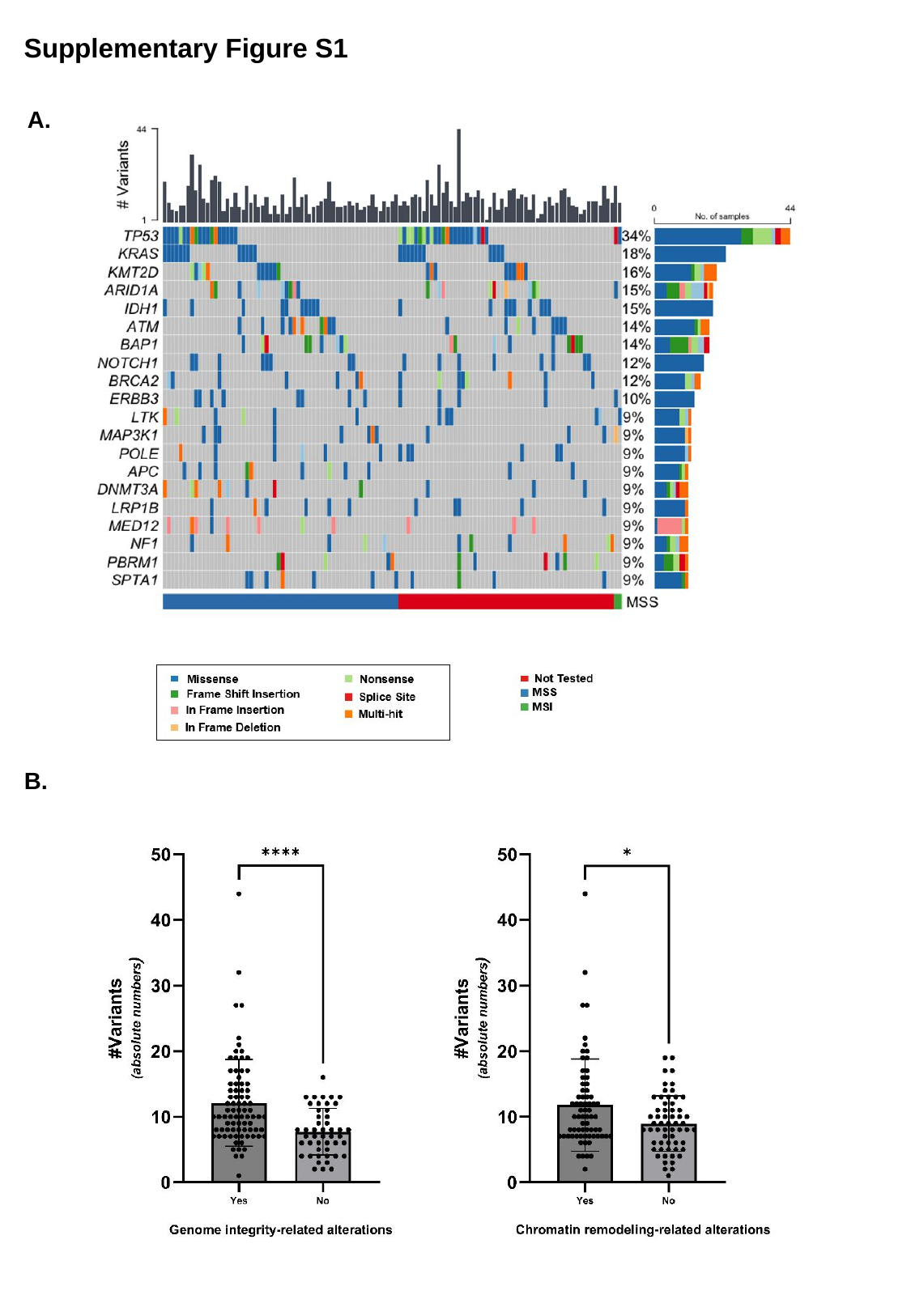

Supplementary Figure S1
A.
B.

## Slide 2
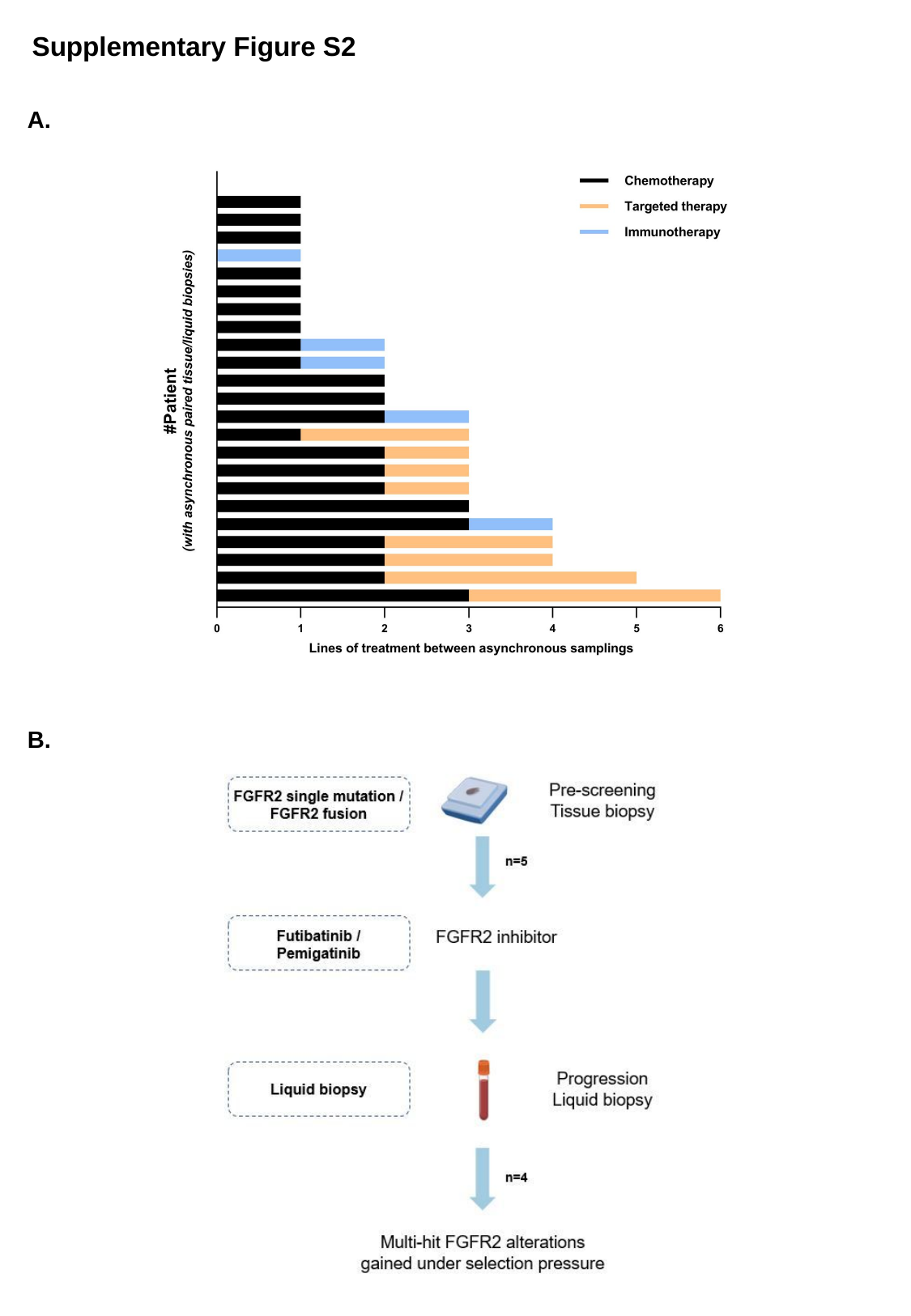

Supplementary Figure S2
A.
B.

## Slide 3
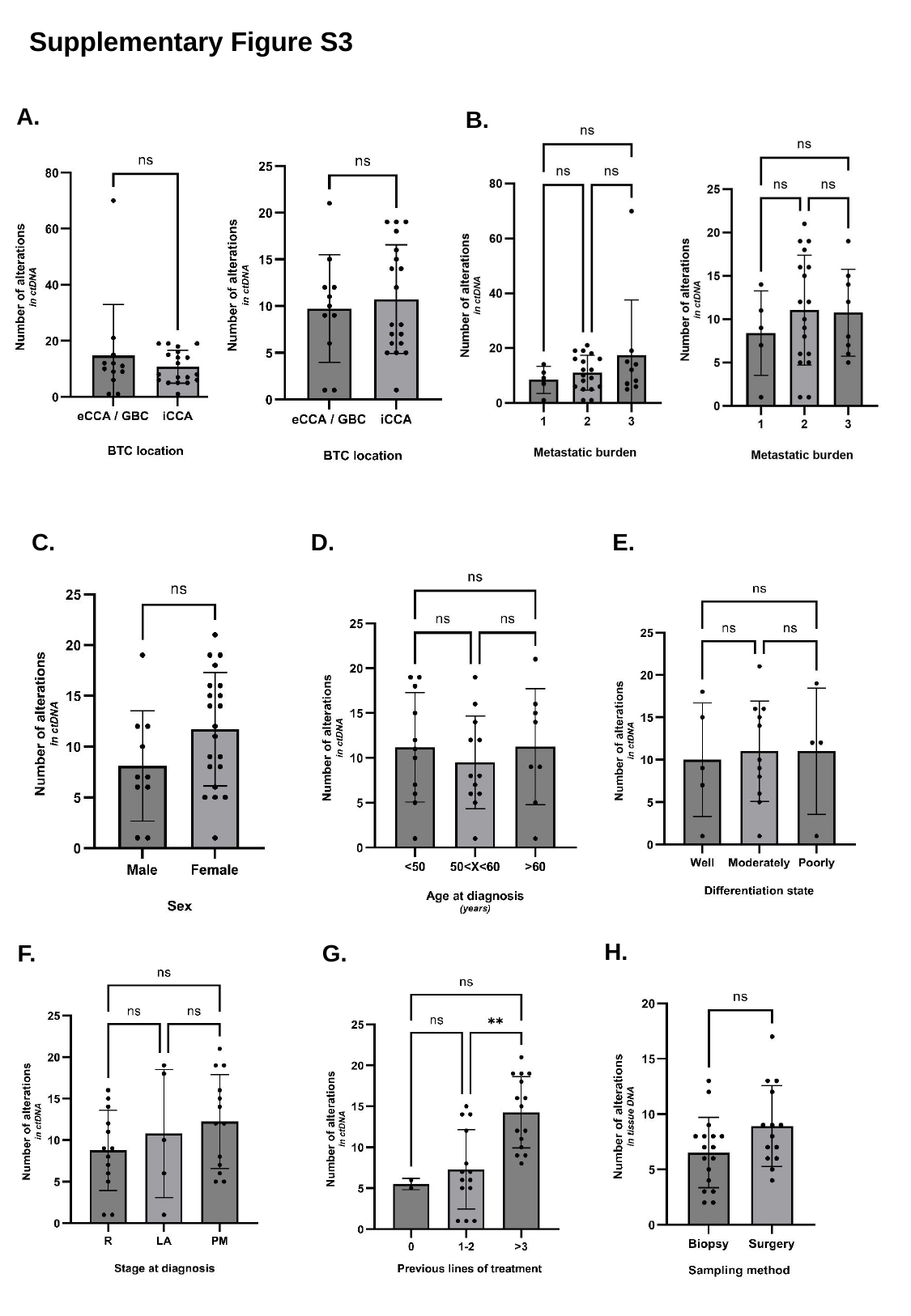

Supplementary Figure S3
A.
B.
C.
D.
E.
H.
F.
G.

## Slide 4
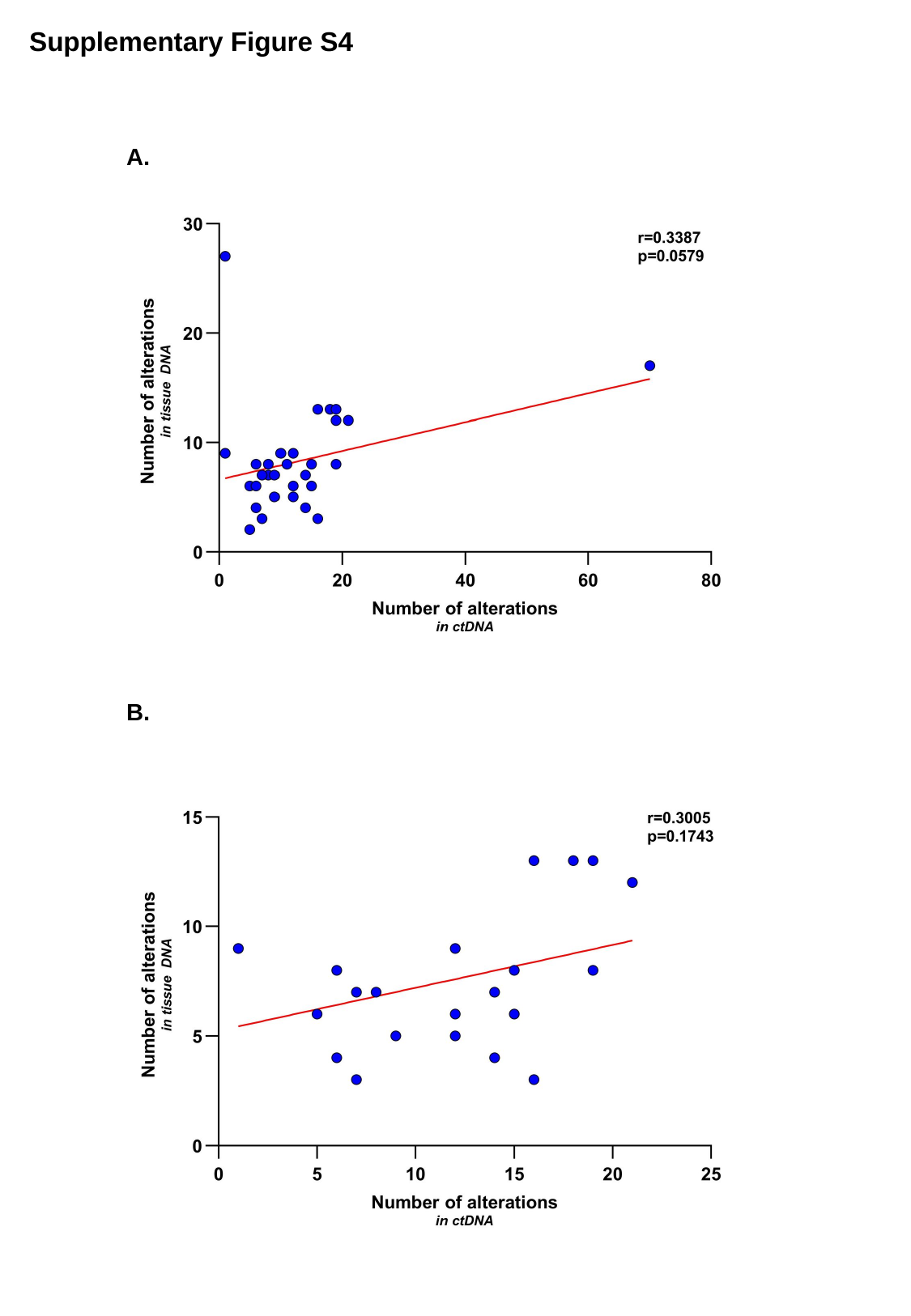

Supplementary Figure S4
A.
B.

## Slide 5
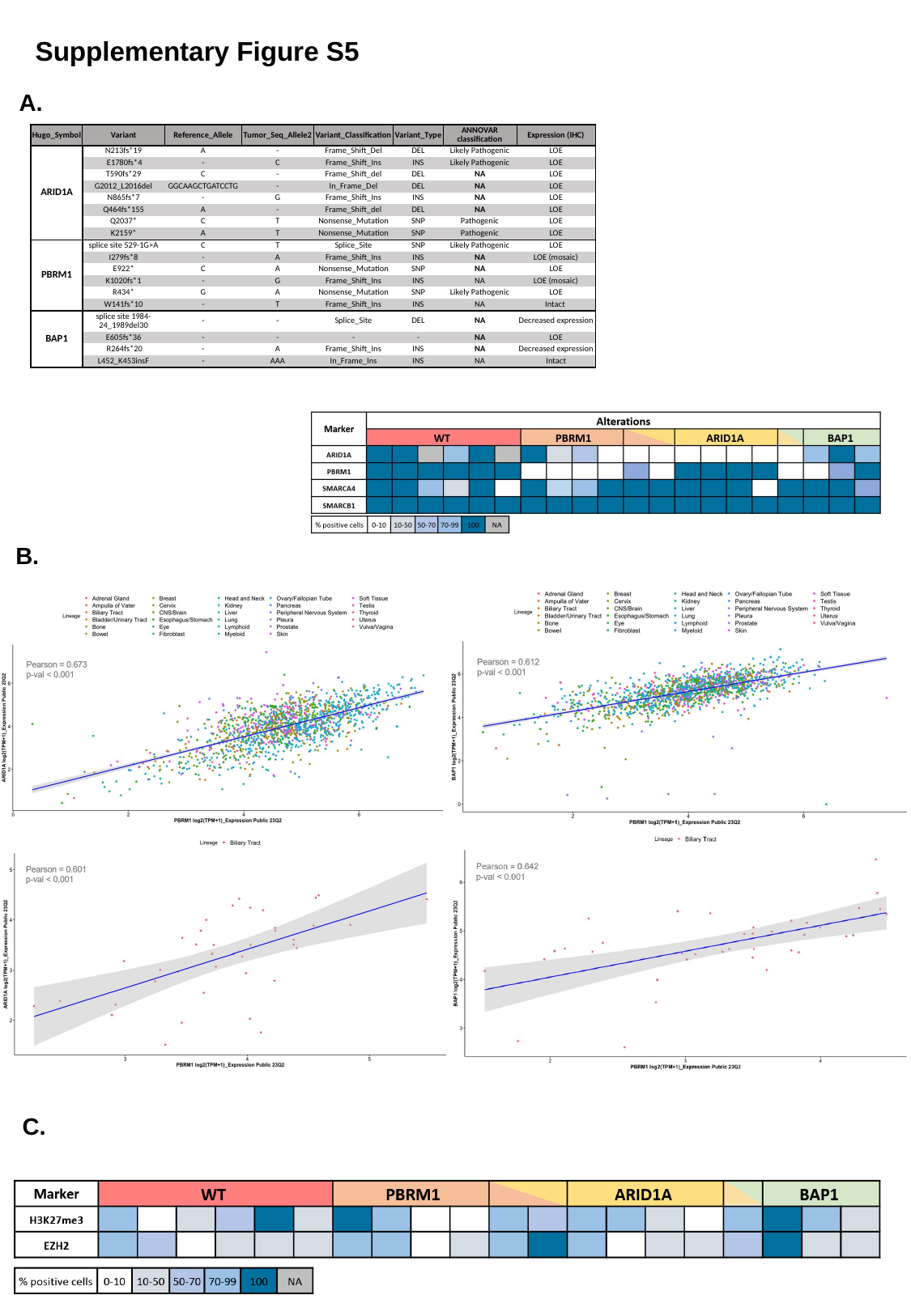

Supplementary Figure S5
A.
| Hugo\_Symbol | Variant | Reference\_Allele | Tumor\_Seq\_Allele2 | Variant\_Classification | Variant\_Type | ANNOVAR classification | Expression (IHC) |
| --- | --- | --- | --- | --- | --- | --- | --- |
| ARID1A | N213fs\*19 | A | - | Frame\_Shift\_Del | DEL | Likely Pathogenic | LOE |
| | E1780fs\*4 | - | C | Frame\_Shift\_Ins | INS | Likely Pathogenic | LOE |
| | T590fs\*29 | C | - | Frame\_Shift\_del | DEL | NA | LOE |
| | G2012\_L2016del | GGCAAGCTGATCCTG | - | In\_Frame\_Del | DEL | NA | LOE |
| | N865fs\*7 | - | G | Frame\_Shift\_Ins | INS | NA | LOE |
| | Q464fs\*155 | A | - | Frame\_Shift\_del | DEL | NA | LOE |
| | Q2037\* | C | T | Nonsense\_Mutation | SNP | Pathogenic | LOE |
| | K2159\* | A | T | Nonsense\_Mutation | SNP | Pathogenic | LOE |
| PBRM1 | splice site 529-1G>A | C | T | Splice\_Site | SNP | Likely Pathogenic | LOE |
| | I279fs\*8 | - | A | Frame\_Shift\_Ins | INS | NA | LOE (mosaic) |
| | E922\* | C | A | Nonsense\_Mutation | SNP | NA | LOE |
| | K1020fs\*1 | - | G | Frame\_Shift\_Ins | INS | NA | LOE (mosaic) |
| | R434\* | G | A | Nonsense\_Mutation | SNP | Likely Pathogenic | LOE |
| | W141fs\*10 | - | T | Frame\_Shift\_Ins | INS | NA | Intact |
| BAP1 | splice site 1984-24\_1989del30 | - | - | Splice\_Site | DEL | NA | Decreased expression |
| | E605fs\*36 | - | - | - | - | NA | LOE |
| | R264fs\*20 | - | A | Frame\_Shift\_Ins | INS | NA | Decreased expression |
| | L452\_K453insF | - | AAA | In\_Frame\_Ins | INS | NA | Intact |
B.
C.
